# Urate Levels as a Predictor of the Prevalence and Level of Cardiovascular Risk Factors: An Identificación de La PoBlación Española de Riesgo Cardiovascular y Renal Study

**DOI:** 10.3390/biom14121530

**Published:** 2024-11-29

**Authors:** Paula Antelo-Pais, Miguel Ángel Prieto-Díaz, Rafael M. Micó-Pérez, Vicente Pallarés-Carratalá, Sonsoles Velilla-Zancada, José Polo-García, Alfonso Barquilla-García, Leovigildo Ginel-Mendoza, Antonio Segura-Fragoso, Facundo Vitelli-Storelli, Vicente Martín-Sánchez, Álvaro Hermida-Ameijerias, Sergio Cinza-Sanjurjo, on behalf of the Investigators of the IBERICAN Study and of the Spanish Society of Primary Care Physicians (SEMERGEN) Foundation

**Affiliations:** 1 Santa Comba Health Centre, Health Area of Santiago de Compostela, PC 15840 Santiago de Compostela, Spain; paula.antelo.pais@sergas.es; 2 Vallobín-La Florida Health Centre, PC 33012 Oviedo, Spain; maprietodiaz@telefonica.net; 3 Fontanars dels Alforins Health Centre, Xàtiva–Ontinyent Department of Health, PC 46635 Valencia, Spain; rafaelmmicoperez@gmail.com; 4 Department of Medicine, Jaume I University, PC 12006 Castellón, Spain; 5 Joaquin Elizalde Health Centre, PC 26004 Logroño, Spain; svelizan@hotmail.com; 6 Casar de Cáceres Health Centre, PC 10190 Cáceres, Spain; jpolog@telefonica.net; 7 Trujillo Health Center, PC 10200 Cáceres, Spain; alfonso.barquilla@gmail.com; 8 Ciudad Jardín Health Center, PC 04007 Málaga, Spain; lginel@gmail.com; 9 Epidemiology Unit, Semergen Research Agency, PC 28009 Madrid, Spain; asegurafr@gmail.com; 10 Gene-Environment-Health Interaction Research Group (GIIGAS)/Institute of Biomedicine (IBIOMED), University of León, PC 24004 Leon, Spain; fvits@unileon.es; 11 Institute of Biomedicine (IBIOMED), Epidemiology and Public Health Networking Biomedical Research Centre (CIBERESP), University of León, PC 24004 Leon, Spain; vicente.martin@unileon.es; 12 Department of Internal Medicine, University Hospital of Santiago de Compostela, PC 15706 Santiago de Compostela, Spain; alvaro.hermida@usc.es; 13 Milladoiro Health Centre, Health Area of Santiago de Compostela, PC 15895 Ames, Spain; scinzas@semergen.es; 14 Health Research Institute of Santiago de Compostela (IDIS), PC 15706 Santiago de Compostela, Spain; 15 Networking Biomedical Research Centre-Cardiovascular Diseases (CIBERCV), PC 28029 Madrid, Spain

**Keywords:** hyperuricemia, cardiovascular risk, primary care

## Abstract

(1) Background: Urate levels lower than the classical cut-off point for defining hyperuricemia can increase cardiovascular risks. The aim of this study is to determine if there is a relationship between different urate levels and classic cardiovascular risk factors (CVRFs). (2) Methods: A cross-sectional study of the inclusion visits of the patients recruited to the IBERICAN study was conducted. The patients were classified into quartiles according to their distribution of urate levels and separated by sex; the three lower points corresponded to normal levels of urate, and the highest quartile was determined according to the classical definition of HU. Multivariate analysis models, adjusted for epidemiological variables, were used to analyze the association of urate levels with CVRFs. (3) Results: The presence of CVRFs was higher across the quartiles of urate, with a continuous increase along the quartiles in both sexes in accordance with body mass index (*p* < 0.01), waist circumference (*p* < 0.01), blood pressure (*p* < 0.01), and LDL cholesterol (*p* < 0.01). The CV risk estimated by SCORE was associated with an increase along the quartiles in women (*p* = 0.02). (4) Conclusions: A progressive increase in the frequency of CVRFs, as well as in their levels, was observed across the quartiles of uricemia, which reflects an increase in the CVRs associated with uricemia.

## 1. Introduction

Atherosclerosis is the pathophysiological basis of cardiovascular disease (CVD). It is a silent process connecting cardiovascular risk factors (CVRFs) to CVD, presenting as irreversible damage. Inflammation plays an important role as a mediator in all the stages of this disease [1].

There is growing evidence that urate may play a pathophysiological role in metabolic disease [2], as well as in the first and second stage of CVD [3]. There are two known mechanisms: inflammation and oxidative stress [4]. However, it also has an antioxidant effect, being responsible for between 50% and 70% of the body’s antioxidant effect [5], acting as an eliminator of waste products such as LDL [6]. Despite the prevalent idea that they may be related to the dysfunction of this antioxidant effect in patients with increased levels of urate [7,8], it is unclear which of these mechanisms is the most important with respect to CVR [8,9].

The accepted classic cut-off values for hyperuricemia (HU) have been defined as urate > 6 mg/dL in women and >7 mg/dL in men based on the urate saturation point and its deposition in the atheromatous plaque; this is specified in the 2018 European Hypertension Guidelines [10]. This definition, based on chemical criteria, has led to confused results between different studies with different cohorts.

Analyses of quartiles differ between studies, with different cut-offs depending on the samples; this may be the reason why the results are different and sometimes contradictory. From the first study, a sub-analysis of the Framingham cohort, which concluded that there was no causal relationship observed [11], to the most recent study, the URRAH study, which concluded that urate is a predictor of total mortality, cardiovascular mortality, and fatal acute myocardial infarction (AMI) [12], there have been many analyses with contradictory results. Some of them have observed an association between elevated levels of urate with CVRFs, such as prehypertension [13], hypertension (HTN) [14], and diabetes mellitus (DM) [15]; others have observed an association with coronary artery disease, stroke [16], and the different subtypes of atrial fibrillation (AF) [17].

Specifically, the analysis of quartiles is infrequent, and the majority of studies only observe a relationship between the highest quartile and subclinical atherosclerosis [18], renal disease [19,20], and cardiovascular mortality [21,22,23], without considering any evidence related to the lower levels. In the same sense that blood pressure and glucose levels increase in intermediate levels that reflect intermediate risk in patients, intermediate levels of urate are potentially associated with moderate risk in patients. Another variable to consider in the HU study with respect to CVR is the influence of each sex. On the one hand, there were differences described in terms of CVRFs [24]; and on the other, there were also differences in the prevalence of AMI [25]. In our opinion, it is necessary to analyze the roles of urate in cardiovascular risk, as there are lower classical levels with respect to CVRFs, as well as how these progress along with urate levels in terms of sex.

The aim of this study is to analyze the association of urate levels with the presence of CVRFs and their respective levels according to sex. Secondly, we analyze the estimated changes in cardiovascular risk along with the different levels of urate.

## 2. Materials and Methods

### 2.1. Study Design

A cross-sectional analysis was performed on patients recruited for the IBERICAN cohort by Spanish primary care physicians (PCPs). This study was approved by the CREC of the Hospital Clínico San Carlos in Madrid on 21 February 2013 (C.P. IBERICAN-C.I. 13/047-E), and the general characteristics of the study have already been published [26].

### 2.2. Patient Selection

The sample of this study consists of 6927 of the 8066 patients of the IBERICAN study from whom urate levels were available. All of them were 18 to 85 years old and were permanently assigned to a PCP [26].

### 2.3. Recorded Variables

Sociodemographic data (sex, age, ethnicity, habitat, level of education, family economic status, current employment status), personal history (HTN, DM, hypercholesterolemia), clinical parameters (weight, height, body mass index (BMI), waist circumference (WC), and systolic and diastolic blood pressure (SBP and DBP)) were collected in the inclusion visit.

HTN was defined as a diagnosis of hypertension, blood pressure levels above 140/90 mmHg, or current use of antihypertensive medication [10]; DM was defined as a diagnosis of diabetes, HbA1c levels above 7%, or use of antidiabetic medication [27]; hypercholesterolemia was defined as a diagnosis of hypercholesterolemia, LDL cholesterol levels above the established thresholds for their risk level, or taking lipid-lowering medication [28].

In addition, the results of blood tests (glucose and cholesterol levels) carried out within the last 6 months were included. Each variable has been defined according to the Clinical Practice Guidelines [26,29].

Finally, CVR stratification of patients was performed according to the SCORE tables for low-risk countries [30].

### 2.4. Statistical Analysis

The qualitative variables were defined as percentages with a 95% confidence interval (95% CI), and the continuous variables were defined as mean with standard deviation (SD) after checking with a Kolmogorov–Smirnov test.

Urate levels were classified by quartiles according to sex with the following cut-off points: Q1 (Men ≤ 4.9 mg/dL; Women ≤ 3.8 mg/dL); Q2 (Men > 4.9–≤ 5.8 mg/dL; Women > 3.8–≤ 4.6 mg/dL); Q3 (Men > 5.8–≤ 6.8 mg/dL; Women > 4.6–≤ 5.5 mg/dL); and Q4 (Men > 6.8 mg/dL; Women > 5.5 mg/dL).

Bivariate analysis was carried out using the Chi-square test for categorical variables, and the ANOVA test was carried out for continuous variables, which was completed with a post hoc analysis (Bonferroni) in cases where statistically significant differences were identified to check which quartiles presented differences.

The means of the continuous quantitative variables (BMI, WC, SBP, DBP, blood glucose, total cholesterol, HDL cholesterol, LDL cholesterol, and triglycerides) were compared with the urate quartiles for each sex using linear regression models, adjusting for age, level of education, physical activity, and adherence to the Mediterranean diet.

For this adjustment, self-reported physical activity was also recorded, and a sedentary lifestyle was defined as less than 30 min of moderate-intensity daily walking for less than 4 days [29]. Adherence to the Mediterranean diet pattern was determined via the Mediterranean Diet Score questionnaire [31].

In all comparisons, the null hypothesis with an alpha error < 0.05 has been rejected. IBM SPSS (Statistical Package for Social Sciences) para Windows (Released 2013, IBM Corp., Armonk, NY, USA), IBM SPSS Statistics for Windows, version 22.0.0.0., IBM Corp., Armonk, NY, USA and STATA 15 (Stata Statistical Software: Release 15, Stata Corp. LLC, College Station, TX, USA) were used for data analysis.

## 3. Results

### 3.1. General Characteristics of the Sample

Mean uricemia levels were 5.88 (1.41) mg/dL in men and 4.71 (1.28) mg/dL in women. Table 1 shows the epidemiological data of patients for the four quartiles; there is an increase in age with each quartile from Q2 (*p* < 0.001), without differences between Q1 and Q2 (*p* = 0.738), and with no other differences in sex or in other variables.

**Figure 1:**
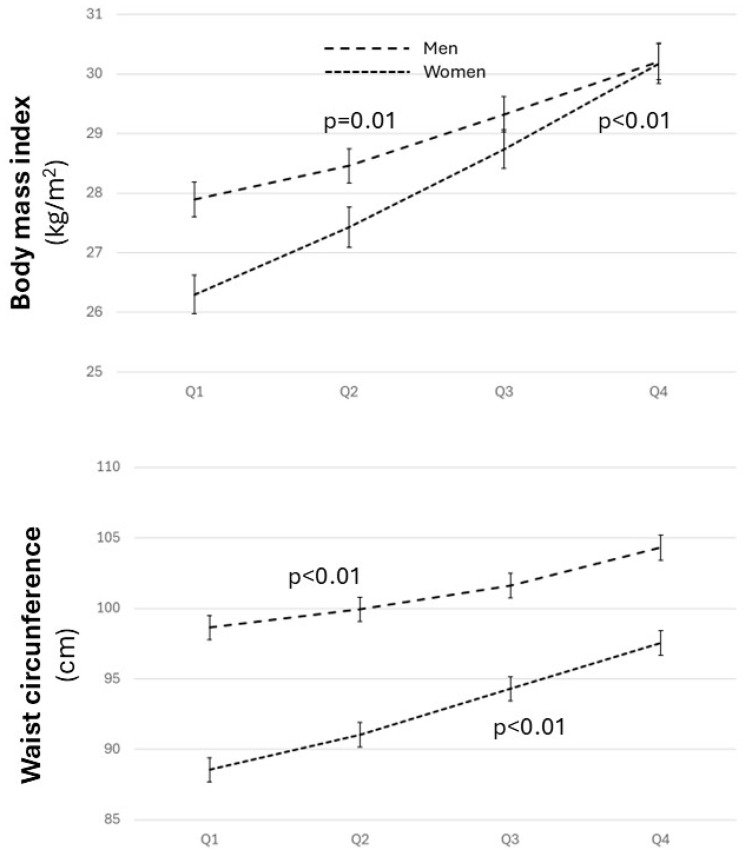
Body mass index and waist circumference across the quartiles for both sexes. ANOVA for the relationship between body mass index and waist circumference in both sexes, adjusted for age, level of education, physical activity, and adherence to the Mediterranean diet.

An increase in BMI and WC was observed across the quartiles in both sexes, although in women, it begins at lower levels in the initial quartiles and increases in both parameters of obesity to reach a similar level to men in the highest quartile (*p* < 0.01) (Figure 1 and Table S1).

**Figure 2:**
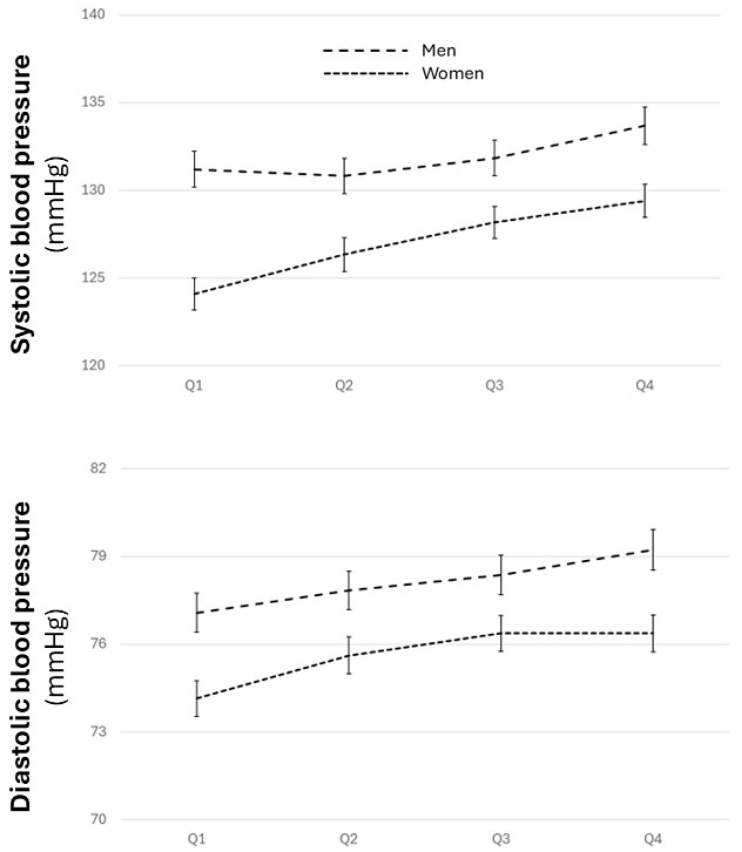
Systolic and diastolic blood pressure across the quartiles for both sexes. ANOVA for the relationship between systolic and diastolic blood pressure in both sexes, adjusted for age, level of education, physical activity, and adherence to the Mediterranean diet.

SBP (*p* < 0.01) and DBP (*p* < 0.01), adjusted for epidemiological variables, showed a progressive increase across the quartiles, with lower levels in women than in men but with a greater increase in lower quartiles in women (Figure 2 and Table S1).

**Figure 3:**
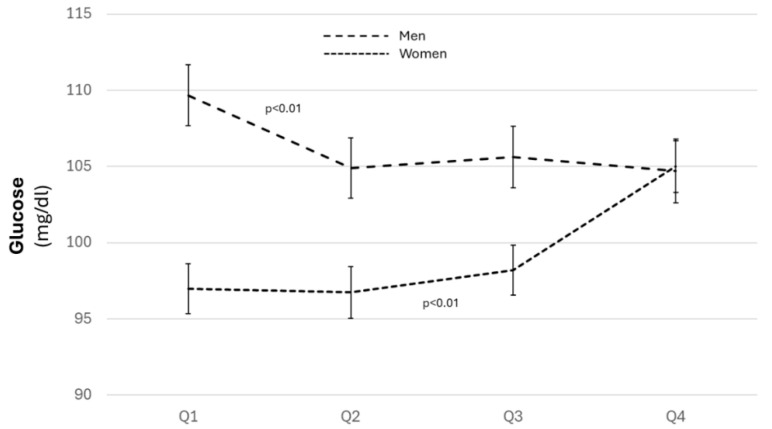
Levels of blood glucose across the quartiles for both sexes. ANOVA for the relationship between blood glucose in both sexes, adjusted for age, level of education, physical activity, and adherence to the Mediterranean diet.

Blood glucose showed changes along the quartiles with different progressions in both sexes; there was an increase in women (*p* < 0.01) and a negative association in men (*p* < 0.01), especially in the highest quartile (*p* < 0.01) (Figure 3 and Table S1).

**Figure 4:**
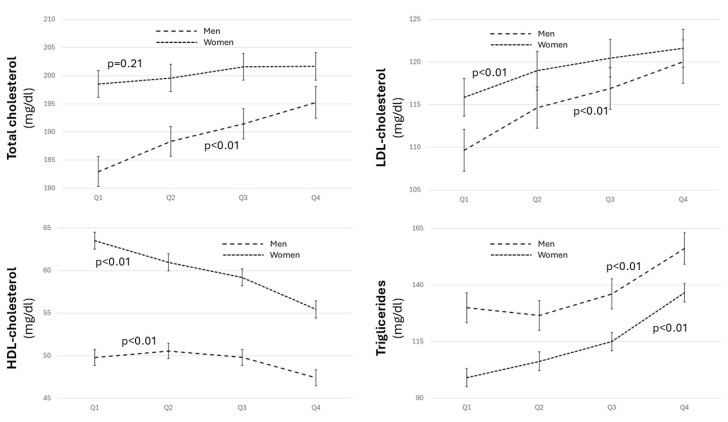
Lipid profile levels across the quartiles for both sexes. ANOVA for the lipid profile relationship in both sexes, adjusted for age, level of education, physical activity, and adherence to the Mediterranean diet.

In both sexes, a similar increase in LDL cholesterol (*p* < 0.01) and triglyceride (*p* < 0.01) levels was observed from Q2 onwards. HDL cholesterol showed a negative relationship in both sexes; however, in women, it was progressive from the lowest quartile; while in men, there was only a significant decrease in the highest quartile (*p* < 0.01) (Figure 4 and Table S1). We did not observe differences in the number of drugs used to combat hypercholesterolemia (0.84 [0.54] vs. 0.79 [0.54] vs. 0.80 [0.54] vs. 0.82 [0.56], *p* = 0.109), and neither did we observe a difference in statins use (being the most frequently employed drug) (31.6% vs. 32.6% vs. 36.8% vs. 30.8%, *p* = 0.591).

### 3.2. Distribution of Cardiovascular Risk Factors and Estimated Cardiovascular Risk

**Figure 5:**
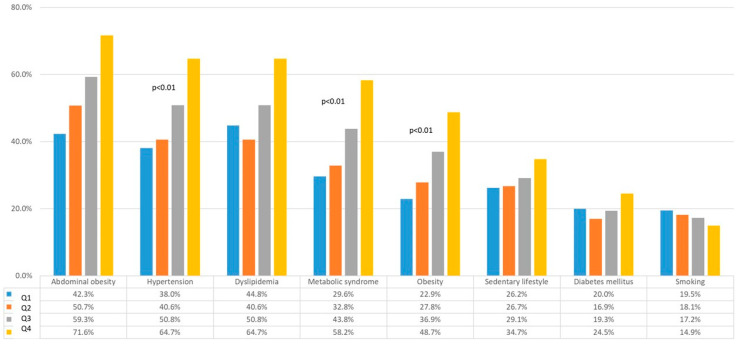
Prevalence of cardiovascular risk factors in each quartile.

Overall, a progressive increase in all CVRFs was observed, parallel to the increase in quartiles, except for smoking, which showed a progressive decline but without statistical significance (*p* = 0.565) (Figure 5). In addition, a different predominance of CVRFs was observed at each level of uricemia: dyslipidemia (44.8%) was the most frequent at the lowest level of uricemia, and abdominal obesity (71.6%) was the most frequent at the highest levels.

**Figure 6:**
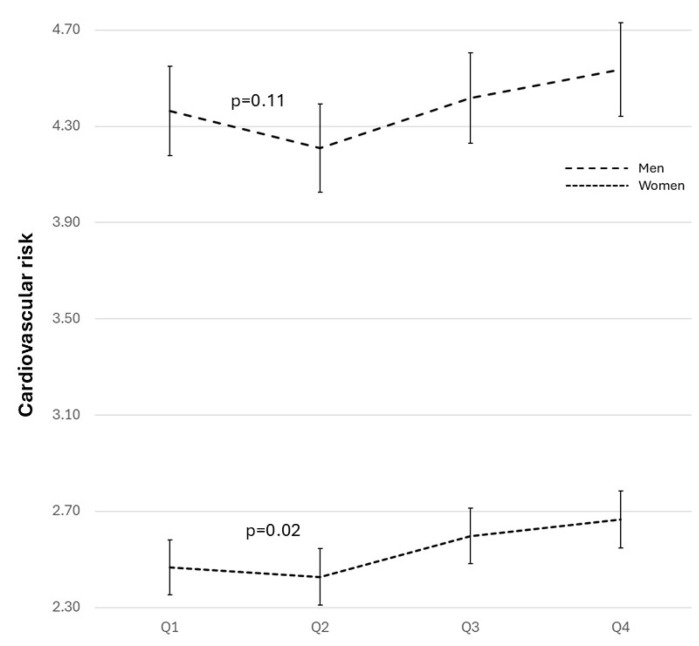
SCORE-estimated cardiovascular risk for both sexes according to the four levels of uricemia. ANOVA for the SCORE risk level relationship in both sexes, adjusted for age, level of education, physical activity, and adherence to the Mediterranean diet.

The CVR estimated by SCORE showed a higher risk in men but without association with urate levels (*p* = 0.11). In women, a progressive increase was observed from Q3 (*p* = 0.02) (Figure 6 and Table S1).

## 4. Discussion

The results obtained from a sample of 6927 patients recruited in primary care indicate that both the prevalence and the levels of each CVRF are progressively higher across the uricemia levels as classified by quartiles, with the exception of glycemia in men. This increase was observed from the lowest levels of urate, with a progressive evolution along the normal levels to eventually reach the classic HU cut-off points. Although we have described CVR associated with HU in previous studies [32], our results expand on these conclusions because there may be an increased risk in the normal uricemia ranges, taking into account the higher prevalence and higher levels of each CVRF. The analysis by sex confirms this: although most of the parameters analyzed are lower in women than men, their increase with increasing uricemia has a better correlation and is more pronounced in women. This could improve the CVR estimation in women and the identification of patients with higher CVR.

Our analyses have divided urate levels by quartiles, bringing the Q4 points very close to the classic definition of HU [10]. This allowed us to analyze the variation in the CVRFs’ prevalence and values along normal urate levels in the three lower quartiles, as well as the classic HU definition in the highest quartile.

Our results describe an association between uricemia and metabolic disorders, such as both parameters of obesity and blood glucose in women, as well as blood pressure and LDL cholesterol in both sexes. These changes were observed from the lowest urate levels and were especially high in the last quartile. All of them began at lower levels for women, but in some of them, such as BMI, blood glucose, and LDL cholesterol, the highest levels in the last quartile were similar to those in men. Our results could indicate that urate reflects the progression along the cardiovascular continuum, with higher values and prevalence of CVRFs along urate levels, and also identify patients with highest CVR as women with metabolic disorders.

The relationship between uricemia and obesity has already been described by other authors in both sexes, proposing a causal relationship between them [33,34] based on the existing linear relationship [35]. Most studies associate obesity, especially abdominal obesity [36], with HU as defined by the dichotomous criteria of the Guidelines [37]. Our results extend this association by using the two most common criteria for obesity and confirming the correlation from normal urate values. In addition, we reinforce the possible synergistic role of obesity and uricemia in the development of other metabolic disorders such as hypercholesterolemia [38], DM [39], or metabolic syndrome [40], with a particular association in women with a higher CVR [41,42]. In the analyses of continuous variables related to cholesterol levels, we observed an increase in total cholesterol, LDL cholesterol, and triglycerides and a reduction in HDL cholesterol across urate quartiles. These results are consistent with the PAMELA study. Maloberti et al. observed an association between the lipid profile and adiposity indexes and HU [43], which could explain the effect of oxidative stress in the lipid profile and the related CVR [44].

Previous studies, such as the TCLSIH (Tianjin Chronic Low-grade Systemic Inflammation and Health) [45] and the STANISLAS (Suivi Temporaire Annuel Non-Invasif de la Santé des Lorrains Assurés Sociaux) studies [13,46], have described a higher incidence of HTN and prehypertension in patients with previous HU. A very interesting recent work observed a relationship between uricemia and non-dipper blood pressure with an increase in CVR [47]. Our results extend this association, describing a progressive increase in blood pressure levels—both systolic and diastolic—in both sexes from very low urate values, which is maintained across the quartiles. Maybe the association between uricemia and HTN could be related to treatment with thiazides, but previous analyses in our sample showed that it was independent [32].

As discussed earlier, these results show that the progressive increase in normal urate levels is the reflection of the first phases of the cardiovascular continuum [48], i.e., when the patient only has CVRFs, and HU levels correspond to the advanced stages of this continuum, with a high prevalence of CVD [32]. Similar results were described in the URRAH study; Maloberti et al. proposed a lower level for defining HU based on their finding of an association between target organ damage (TOD) and lower urate levels [49].

Our results also showed differences between sexes. We found a high impact of urate levels in women, with a high correlation with some CVRFs, as well as a higher correlation between CVR and urate levels. There are no studies that analyze this association, but some works describe a higher association between urate levels and CVD in women than in men, such as the URRAH study [25] or Sun et al. [50].

### Strengths and Limitations

Our results are statistically robust and consistent with the existing literature on this topic. The sample size and the recruitment of patients in primary care gives our work sufficient statistical power to answer the research question posed, with good representativeness of the sample and generalization of the results, which would allow for the better applicability of the results to a larger number of patients. The sex-disaggregated analysis confirms differences in the association of urate with cardiovascular risk factors, sometimes differing from those described in the general sample, and thus enabling us to draw more accurate conclusions.

As said before, our overall results are consistent with the published literature, but we have contributed important knowledge by describing a progressive increase in all of the analyzed cardiovascular risk factors across urate values which are usually considered normal. This could reflect an increased cardiovascular risk throughout the distribution of these values.

However, our study also has limitations. Some have been described in previous publications [48,51], but others should be mentioned here due to their relationship with this work. On the one hand, the analyses were not carried out in a centralized laboratory, and the values used for the analysis were those provided by the reference laboratories of each health center participating in the study; in any case, however, they are all laboratories of the Spanish National Health System, which reflect the usual clinical practice. We should also point out that patients receiving urate-lowering treatments have not been excluded. This would classify patients who actually have HU as “healthy”, who may have a higher incidence of events than actually healthy patients. Thus, the differences we describe could be greater, which would confirm our conclusions. In spite of these limitations, and taking into account that the results have been obtained in a large sample of patients who were followed up by their PCP, it should be noted that our findings show the association of uricemia with CVRFs from very low urate levels, i.e., lower than those considered HU. 

On the other hand, some might miss an analysis with other diseases, such as gout or metabolic syndrome. But metabolic syndrome is defined by the coexistence of risk factors that we have included in our results; if our results showed an increase along with urate levels, a progressive increase in all of them can be expected [49]. The diagnosis of gout, although associated with cardiovascular risk, is more a consequence of usually elevated urate levels than an association with cardiovascular risk or risk factors. An adjustment of our model to include gout would only neutralize the effect of elevated urate levels as it is at this level that the highest number of gout episodes occur [21].

This relationship broadens the concept of CVR associated with HU, in which there is a higher incidence of CV events [51]. It also highlights the possibility that urate levels below HU diagnostic values should be included as an estimation variable for CVR. This would give uricemia a role as a facilitator or an enhancer of CVR in patients with CVRFs. In any case, this prognostic association should be confirmed in prospective studies that analyze their incidence.

## 5. Conclusions

We can conclude that there is an association between the most classic CVRFs and uricemia, beginning from very low urate levels, and that this association is progressive and positive across all levels of uricemia. This has been seen in both sexes, although it could have a greater impact on women, especially at higher urate values.

In the CVR study, our results show that urate levels can reflect the stage of the cardiovascular continuum where the patient is, i.e., lower levels reflect its beginning, with presence of CVRFs, which increase progressively to higher levels, associated with subclinical target organ damage, and, finally, to HU levels that lead to CVD.

Longitudinal studies are needed in order to analyze the incidence of cardiovascular and renal events across the range of urate values, providing more information in this regard.

## Figures and Tables

**Table 1:** Sociodemographic characteristics of patients in each quartile.

	Q1		Q2		Q3		Q4		*p*
	1609		1821		1697		1800		
	N	Mean [SD]	N	Mean [SD]	N	Mean [SD]	N	Mean [SD]	
**Age (years)**	1609	55.91 [14.94]	1821	56.91 [15.06]	1697	59.55 [14.14]	1800	62.15 [13.55]	<0.001
	**N**	**%**	**N**	**%**	**N**	**%**	**N**	**%**	
**Women**	873	54.3%	993	54.5%	878	51.7%	995	55.3%	0.136
**Ethnicity**									
White	1545	96.0%	1747	95.9%	1641	96.7%	1745	96.9%	0.656
Black	4	0.2%	7	0.4%	7	0.4%	12	0.7%	
Latin American	49	3.0%	55	3.0%	41	2.4%	34	1.9%	
Asian	2	0.1%	1	0.1%	1	0.1%	3	0.2%	
Berber	9	0.6%	11	0.6%	7	0.4%	6	0.3%	
**Habitat**									
Urban	947	58.9%	1056	58.0%	999	58.9%	1059	58.8%	0.986
Semi-urban	342	21.3%	398	21.9%	353	20.8%	381	21.2%	
Rural	319	19.8%	367	20.2%	344	20.3%	360	20.0%	
**Education**									
Unschooled	116	7.2%	126	6.9%	160	9.4%	225	12.5%	0.124
Primary education	863	53.6%	1014	55.7%	931	54.9%	1006	55.9%	
Higher education	377	23.4%	430	23.6%	376	22.2%	378	21.0%	
University education	253	15.7%	251	13.8%	230	13.6%	191	10.6%	
**Employment**									
Jobholder	739	46.0%	810	44.6%	718	42.5%	647	36.0%	0.138
Unemployed	141	8.8%	160	8.8%	143	8.5%	125	7.0%	
Retired	493	30.7%	609	33.6%	617	36.5%	766	42.7%	
Student	23	1.4%	29	1.6%	15	.9%	9	.5%	
Housekeeping	209	13.0%	207	11.4%	197	11.7%	248	13.8%	
**Income**									
Annual income lower than 18,000 EUR	664	41.3%	744	40.9%	719	42.4%	793	44.1%	0.935
Annual income between 18,000 EUR and 100,000 EUR	927	57.6%	1057	58.0%	953	56.2%	983	54.6%	
Annual income higher than 100,000 EUR	18	1.1%	20	1.1%	25	1.5%	24	1.3%	

Quantitative variables are presented as mean [SD]; qualitative variables are presented as %.

## Data Availability

The authors may provide the data used for the development of this article upon a justified request addressed to the corresponding author.

## Supplementary Materials

The following supporting information can be downloaded at https://www.mdpi.com/article/10.3390/biom14121530/s1, Table S1: Numeric parameters of the relationship between cardiovascular risk factors and urate quartiles.

## Appendix A

The investigators of the IBERICAN study and of the Spanish Society of Primary Care Physicians (SEMERGEN) Foundation:

**Scientific Committee:** Alfonso Barquilla García; Ángel Díaz Rodríguez; Carlos Escobar Cervantes; Francisco Javier Alonso Moreno; Jesús Vergara Martín; Juan José Badimón; José Polo García; Luis Rodríguez Padial; Miguel Ángel Prieto; Rafael Vidal Pérez; Sergio Cinza Sanjurjo; Sonia Miravet Jiménez; Sonsoles Velilla Zancada, José Ramón Banegas, Vicente Martín Sánchez, Vicente Pallares Carratalá, Antonio Segura Fragoso y Rafael Manuel Micó Pérez.

**Researchers: Andalusia:** Antonio López Téllez, Jesus Vergara Martin, Maria De Los Angeles Ortega Osuna, Cristóbal Prieto Cid, Mª José Hidalgo Fajardo, Jose Lorente Serna, Ángel Domínguez Requena, Ricardo Alberola Cañizares, Manuel Ruiz Peña, Filomena Herrero Collado, Marcela Montes Vázquez, Rafael Ángel Carrascal Garrido, Maria Reyes Herrera Lozano, Beatriz Ortiz Oliva, Francisco José Anguita, Carmen Pérez Ibáñez, Carlos Alberto Cabrera Rodríguez, Maria Jose Cruz Rodríguez, Sandra Bonilla Ruiz, Rocio Reina Gonzalez, Salome Abad Sanchez, Inmaculada Santana Martinez, Rafael Sanchez Jordán, Juan Mª Ramos Navas-Parejo, Jose Manuel Ramirez Torres, José María Beltrán Poveda, María Adoración De Cruz Benayas, Carmen Fernandez Gil, Jon Iñaki Esturo Alcaine, Antonio Mora Quintero, Fernando Leiva Cepas, José Luis Carrasco Martín, Emilio Garcia Criado, Mercedes Vazquez Blanco, Isabel Mora Ortiz, Leovigildo Ginel Mendoza, Juan Carlos Aguirre Rodriguez, Esperanza María Romero Rodríguez, Jose Acevedo Vazquez, Juan Gabriel García Ballesteros, María De La Paz Fernández Lara, Patricia Agüera Moreno, Eduardo Paños Maturana, Juan Manuel Ignacio Expósito, Noelia Carrillo Peñas, Carmen María Abad Faya, Ana Marina Almagro Duque, Rubén Torrescusa Camisón, Paloma Menéndez Polo, Marina Peña García, Cristina Lopez Fernández, Ascensión Estepa Torres, Miguel Gutiérrez Jansen, Esperanza Loizaga González, Lisardo Garcia Matarin, Enrique José Gamero De Luna, Javier Benítez Rivero, Maria Jose Gomez Gonzalez, Carmen Gómez Montes, Juan Carlos Rodríguez Rodríguez, Juana María González Barranco, Josefa Ramírez Vizcaíno, María Ángeles Miranda Sánchez. **Aragon:** Eva Trillo Calvo, Concepción Bayod Calvo, Susana Larripa De La Natividad, German Grasa Lambea, Emilio Jiménez Marín, Ana Cristina Navarro Gonzalvo, Antonio Pablo Martinez Barseló, Irene Peña León, Ángel González Pérez, Liliana Mahulea. **Asturias:** María José Pérez Martínez, Ana Piera Carbonell, Margarita Alonso Fernández, María Montserrat Rueda Cuadrado, Rodrigo Abad Rodríguez, José Miguel Alvarez Cabo, Rubén Sánchez Rodríguez, Eva Maria Cano Cabo, Anny Romero Secin, Miguel Ángel Prieto Diaz, Juan Jesús García Fernández, Saul Suárez García. **Balearic Islands:** Fernando García Romanos, Antonia Moreno Gonzalez, Maria Lara Amengual Sastre, Susana Martínez Palli, José Alfonso Ramón Bauza, Jose Ortiz Bolinches, Carmen Fernandez Fernández, Maria Isabel Orlandis Vázquez, Ana Sanchis Mezquita, Fernando Unceta Aramburu, Juan Fernando Peiró Morant, Ana Moyá Amengual, Mateu Seguí-Díaz. **Basque Country:** Jose Felix Zuazagoitia Nubla, Ana Echevarría Ituiño, Gregorio Mediavilla Tris, María Carmen Noriega Bosch, Esther Gonzalez, Maria Luisa Ruiz Macho, Ruth Sendino Del Olmo, Asunción Olagorta De Prado, Ana López De Viñaspre Muguerza, Jesús Iturralde Iriso, Mª Rosario Virtus Iñurrieta, Lucas Ulloa Bahamonde. **Canary Islands:** Isidro Godoy Garcia, Fernando Rubio Sevillano, María Isabel González González, Marta Pérez Souto, Raquel De León Contreras, Sara Isabel Almeida González, Irene Almería Diez, Virginia Maria Mirabal Sánchez, Francisco Jose Escobar Lavado, Yoel Anta Pérez, Nayra Sánchez Hernández, Juan Luis Alonso Jerez, Ricardo Koch, Nayra Ramirez Mendoza, Héctor Suárez Hernández, Francisco Jesús Morales Escobar. **Cantabria:** E. Lidia Gutiérrez Fernández, Fernando Andrés Mantecón, Ana Belen Garcia Garrido, Asuncion Velez Escalante, Luisa Alonso Rentería, Jesús Sainz Jiménez, Guillermo Pombo Alles, Esperanza Rueda Alonso. **Castilla La Mancha:** Juan Antonio Divison Garrote, Pedro Martínez Sotodosos, Juan Antonio Vivancos Fuster, María García Palencia, José Ambrosio Torres Moraleda, Sara González Ballesteros, Ana Carmen Gil Adrados, Antonio González Cabrera, Miguel Angel Babiano Fernandez, Guillermo Rico García, Juan Jose Criado-Alvarez, Pilar Torres Moreno, Francisco Javier Arribas Aguirregaviria, Alicia Sahuquillo Martínez, Lourdes Maria Santos Bejar, Miguel Laborda Peralta, Raul Piedra Castro, Carlos Santos Altozano, Lucia González-Tarrio Polo, Pedro Valiente Maresca, Reinilda Mota Santana, Noemi Elizabeth Terrero Ledesma, Noelia Garrido Espada, Francisco Javier Alonso Moreno, Gabriela Delia Rosa Zambrana Calvi, Cristina de Castro Mesa, Blanca Cordero García, Pilar Sorrius Sitges, Ana María de Santiago Nocito, César Lozano Suárez. **Castilla y León:** Juan Lorenzo Gutierrez Montero, Juan Ignacio López Gil, Maria Dolores Fernández Ortega, Miren Elizari Roncal, María Ascensión López Serrano, Nuria Esther Adrian De La Fuente, Belén Angulo Fdez. De Larrea, Naiara Cubelos Fernández, Guiomar Luz Ferreiro Gómez, Diana Gomez Rodriguez, Sonia María Andrés Tuñón, María Ajenjo González, Serafín De Abajo Olea, Juan Jose Leon Regueras, César Manuel Gallego Nieto, Delio Vazquez Mallada, Maria De La O Gutierrez Garcia, Pablo Baz Rodríguez, José Ignacio Ferradal García, Blanca Delia De Román Martínez, Ana Arconada Pérez, Omar Mahmoud Atoui, Álvaro Morán Bayón, María Teresa Armenteros Del Olmo, Fco Javier García-Norro Herreros, Enrique Méndez Rodríguez, Diana María Narganes Pineda, Ángel Díaz Rodríguez, Verónica Ortiz Ainaga, Milagros Sonlei Sánchez Guevara, Laura Villota Ferreiro, M Teresa Grande Grande, Francisco Vicente Martínez Gracía, Jesús Palomo del Arco. **Catalonia:** María Dolores Moriano García, Beatriz Jiménez Muñoz, Gemma Rovira Marcelino, Diana Elizabeth Fernandez Valverde, Roser Rodó Bernadó, Maria Teresa Ortiz Lupiañez, Najlaa Najih, José María Diéguez Parra, Mª Rosa Benedicto Acebo, Mari Luz Bravo Vicien, Alberto Ramón León Estella, Juan Antonio Muñoz Gómez, Alicia Mostazo Muntané, Isabel Ortega Abarca, Anna Gasol Fargas, Brenda Elizabeth Riesgo Escudero, Susana Elizabeth Riesgo, Edgar Zaballos Castellvi, Celia Cols Sagarra, Marta Herranz Fernández, Josep Alins Presas, Idaira Damas Pérez, Rosa M Alcolea Garcia, Ines Monte Collado, Roberto Genique Martínez, María José Guasch Villanueva, Sònia Miravet Jiménez, Teresa Rama Martinez, Lucio Pinto Pena, Josefa Maria Panisello Royo, Inés Gil Gil, Carlos Gómez Ruiz, Rita Sahun Font, Anna Fuentes Lloveras. **Community of Madrid:** Alberto Calderon Montero, María Del Mar Zamora Gómez, Elena Alarcón Cebrián, Mª José Piñero Acin, Celia Pecharroman Sacristán, M Soledad Mayayo Vicente, Mª Paz Pérez Unanua, Nuria Marañón Henrich, Saray Gómez Monreal, Sonia Redondo De Pedro, Blanca Sanz Pozo, Irene Moreno Martinez, Beatriz Lopez Uriarte, Carmelina Sanz Velasco, Amaya Gárriz Aguirre, Montserrat Rivera Teijido, German Reviriego Jaén, Jose Ignacio Aza Pascual-Salcedo, Josefa Vázquez Gallego, Julia Caballer Rodilla, Aida Herrera, Ezequiel Arranz Martínez, Ana María Gómez Calvo, Paula Morán Oliva, Mª Milagros González Béjar, Julio Antonio Heras Hitos, Olga Garcia Vallejo, Manuel De Jesús Frías Vargas, María Jesús Castillejo Boguerin, Aurora García Lerín, Miguel Angel María Tablado, Elena Concepción García García, Leticia De Miguel Acero, Carmen Zárate Oñate, Aránzazu Barranco Apoita, María Ester Montes Belloso, Ana Maria Huertas Velasco, Rafael Sáez Jiménez, Julia Natividad Garcia Pascual, María Clemencia Zuluaga Zuluaga, Mª Cruz Díez Perez, Antonio Ruiz García, Cristina Murillo Jelsbak, Virginia Lasso Oria, Amelia Gonzalez Gamarra, Elena Rodilla Rodilla, Alberto Galgo Nafría, María Mestre de Juan, Mª Carmen García Albiñana, Mª del Pilar Moreno Cano, Paula Hernanz López, Paloma Casado Pérez. **Extremadura:** Jacinto Espinosa García, Jose Ignacio Prieto Romo, Leandro Fernández Fernández, Javier Sierratapia, Nieves Moreno Regidor, Francisco Javier Zaballos Sánchez, Ana Moreno Moreno, Francisco Carramiñana Barrera, Juan Jose Torres Vazquez, Maria José Gamero Samino, Miguel Angel De Santiago Rodriguez, Pablo Rafael Gómez Martínez, Antonio Carlos Elias Becerra, Javier Soto Olivera, Víctor Cambero, Julián Domínguez Ávila, Andrés Simón Fuentes, Jorge Manuel De Nicolas Jimenez, Dimas Igual Fraile, Guadalupe Nieto Barco, Ignacio Araujo Ramos, Mª Luz Serrano Berrocal, Francisco Buitrago Ramírez, Minerva Gallego Marcos, Felix Suarez Gonzalez, Victoriano Chavero Carrasco, José Polo García, Francisco Guerra Peguero, Francisco Javier Sanchez Vega, Manuel Tejero Mas, Alba Palmerín Donoso, Miguel Turégano Yedro, María Beatriz Esteban Rojas, Fátima Cabezudo Moreno, Nawson Elver Quevedo Saldaña, Maria Del Mar García Fenés, Alfonso Barquilla García, Timotea Garrote Florencio, Jose Maria Fernandez Toro, Vicente Caballero Pajares, María José Gómez Barquero. **Galicia:** Alejandra Rey Rañal, Elena García Del Río, Enrique Nieto Pol, Julio Álvarez Fernández, Pilar Alonso Álvarez, Mª Luisa Jorge Gómez, Antonio Calvo Guerrero, Isabel Celemín Colomina, Lucia Barreiro Casal, Juana Fernandez Moreno, Mª Angelines Carballal Martinez, Nabor Diaz Rodriguez, Carlos Moral Paredes, Dolores Recarey García, Fco Javier Iglesias Mato, Antonio Fouz Ulloa, Amparo Fidalgo Gonzalez, Noelia Dios Parada, Patricia Conde Sabarís, Ana Isabel Rodriguez Pérez, Ana Inés García Palacio, Victor Julio Quesada Varela, Lidia Romero Iglesias, Ángel Lado Llerena, Carmen Lires Rodríguez, Maria Luisa Carretero Díaz, José Carreira Arias, José Luís Vázquez Camino, María Del Carmen Torreiro Penas, Sandra Yáñez Freire, Sergio Cinza Sanjurjo, Daniel Rey Aldana, Carlos Piñeiro Díaz, Portal González Lorenzo, José Rodríguez Campos, Rubén Blanco Rodríguez, Manuel Portela Romero, Lucía Vilela de Castro. **La Rioja:** Sonsoles María Velilla Zancada, Rafael Crespo Sabarís, Oscar Fernando Isaula Jiménez. **Melilla:** Jesus Manuel Gonzalez Puga, Jorge Antonio Benaín Ávila, Óscar Del Toro González. **Murcia:** Vicente Llorca Bueno, Ana María Ballesteros Pérez, Domingo J. Rubira López, Mª Dolores Esteve Franco, Elena Sánchez Pablo, Maria Teresa Palacios López, Juan Castillo Meroño, José María Lobo Martinez, Isabel Maria Peral Martinez, J. Eduardo Carrasco Carrasco, Armando Santo González, Juan Gomariz García, Beatriz Ríos Morata. **Navarra:** Laura Sánchez Iñigo, Ines Sanz Perez. **Valencian Community:** Vicente Pascual Fuster, Mª Dolores Aicart Bort, Natividad Vázquez Gómez, Carlos Lluna Gasco, Teresa Amoros Barber, Pedro Antonio Medina Cano, Miguel Monteagudo Moncho, Mª Jesús Larré Muñoz, Raquel Navarro Hernández, Francisco Jose Martinez Egea, Antonio Tramontano, Marta Ferrer Royo, Belén Persiva Saura, Juan A. Contreras Torres, José Mª Tirado Moliner, Alejandro Salanova Penalba, Ariadna Cucó Alberola, Fernando Maria Navarro I Ros, Enrique Beltrán Llicer, Ana Seoane Novás, Inmaculada Martín Valls, Gracia Verdú Mahiques, Enrique Peña Forcada, Nieves Aguilar Gómez, Francisco Javier Sanz García, M Dolores Paradís Bueso, María Eugenia Alegre Romero, Antonio Francés Camus, María Amparo Anton Peinado, Rosa Latorre Santos, Mª Asuncion Palomar Marin, Maria Carmen Botella García, Eva Sánchez Fresquet, Pedro Sala Paños, Tomás Sánchez Ruiz, Rosa Ana Valero Valero, María Seoane Vicente, Magdalena Martin Llinares, Antonio Masiá Alegre, José Luis Llisterri Caro, Irene Lluch Verdu, Vicente Pallares Carratala, Francisco Valls Roca, Rafael Manuel Micó Pérez, Carmen Barcelo Dupuy, Elena Benages Vicente, María José Gimeno Tortajada, Mercedes Calleja del Ser, Martín Menéndez Rodríguez, Rosalía Victoria Carbonell Castelló.
