# Supplementary material for: Urate Levels as a Predictor of the Prevalence and Level of Cardiovascular Risk Factors: An Identificación de La PoBlación Española de Riesgo Cardiovascular y Renal Study
Source: Biomolecules. 2024 Nov 29;14(12):1530. doi: 10.3390/biom14121530 (PMC11673880; doi:10.3390/biom14121530)
Supplement: Supplementary file 1 [file biomolecules-14-01530-s001.zip › biomolecules-3269908-supplementary.pdf]

Supplementary Materials

Table S1. Numeric parameters of the relationship between cardiovascular risk factors and urate quartiles.

Variables		Q1				Q2			
		Coefficient	95% CI		*p*	Coefficient	95% CI		*p*
BMI	Men	27.9	27.6	28.2	<0.001	28.5	28.2	28.8	<0.001
	Women	26.3	26.0	26.6	<0.001	28.7	28.4	29.1	<0.001
WC	Men	98.6	97.8	99.5	<0.001	99.9	99.1	100.8	<0.001
	Women	88.6	87.7	89.4	<0.001	91.1	90.2	91.9	<0.001
SBP	Men	131.2	130.2	132.2	<0.001	130.8	129.8	131.8	<0.001
	Women	124.1	123.2	125.0	<0.001	126.3	125.4	127.3	<0.001
DBP	Men	77.1	76.4	77.7	<0.001	77.8	77.2	78.5	<0.001
	Women	74.1	73.5	74.8	<0.001	75.6	75.0	76.3	<0.001
Glucose	Men	109.7	107.7	111.7	<0.001	104.9	102.9	106.9	<0.001
	Women	97.0	95.3	98.6	<0.001	96.7	95.0	98.4	<0.001
Total Cholesterol	Men	183.0	180.3	185.6	<0.001	188.3	185.7	191.0	<0.001
	Women	198.5	196.2	200.9	<0.001	199.6	197.1	202.0	<0.001
LDL-cholesterol	Men	109.6	107.2	112.1	<0.001	114.7	112.2	117.1	<0.001
	Women	115.8	113.7	118.0	<0.001	119.0	116.7	121.3	<0.001
HDL-cholesterol	Men	49.8	48.9	50.7	<0.001	50.6	49.6	51.5	<0.001
	Women	63.5	62.5	64.5	<0.001	61.0	59.9	62.0	<0.001
Triglycerides	Men	130.0	123.4	136.6	<0.001	126.5	120.0	133.1	<0.001
	Women	99.1	95.0	103.1	<0.001	106.2	102.1	110.4	<0.001
SCORE	Men	4.4	4.2	4.6	<0.001	4.2	4.0	4.4	<0.001
	Women	2.5	2.4	2.6	<0.001	2.4	2.3	2.5	<0.001
**Variables**		**Q3**				**Q4**			
		**Coefficient**	**95% CI**		*p*	**Coefficient**	**95% CI**		*p*
BMI	Men	29.3	29.0	29.6	<0.001	30.2	29.9	30.5	<0.001
	Women	28.7	28.4	29.1	<0.001	30.2	29.8	30.5	<0.001
WC	Men	101.6	100.7	102.5	<0.001	104.6	103.4	105.2	<0.001
	Women	94.3	93.4	95.1	<0.001	97.5	96.7	98.4	<0.001
SBP	Men	131.8	130.8	132.9	<0.001	133.7	132.6	134.7	<0.001
	Women	128.2	127.2	129.1	<0.001	129.4	128.4	130.3	<0.001
DBP	Men	78.4	77.7	79.0	<0.001	79.2	78.5	79.9	<0.001
	Women	76.4	75.8	77.0	<0.001	76.4	75.7	77.0	<0.001
Glucose	Men	105.6	103.6	107.7	<0.001	104.7	102.6	106.8	<0.001
	Women	98.2	96.6	99.8	<0.001	105.0	103.3	106.7	<0.001
Total Cholesterol	Men	191.4	188.7	194.1	<0.001	195.2	192.4	198.0	<0.001
	Women	201.6	199.2	203.9	<0.001	201.7	199.2	204.1	<0.001
LDL-cholesterol	Men	116.9	114.4	119.3	<0.001	120.0	117.5	122.6	<0.001
	Women	120.5	118.3	122.6	<0.001	121.6	119.4	123.9	<0.001
HDL-cholesterol	Men	49.8	48.9	50.7	<0.001	47.4	46.4	48.4	<0.001
	Women	59.2	58.2	60.2	<0.001	55.4	54.4	56.4	<0.001
Triglycerides	Men	136.1	129.4	142.8	<0.001	156.1	149.2	163.1	<0.001
	Women	115.0	110.9	119.0	<0.001	136.6	132.5	140.8	<0.001
SCORE	Men	4.4	4.2	4.6	<0.001	4.5	4.3	4.7	<0.001
	Women	2.6	2.5	2.7	<0.001	2.7	2.5	2.8	<0.001

BMI: body mass index; WC: waist circumference; SBP: systolic blood pressure; DBP: diastolic blood pressure.
